# Disseminated Tuberculosis of the Axial Skeleton

**DOI:** 10.4269/ajtmh.23-0691

**Published:** 2024-02-20

**Authors:** Gawahir A. Ali, Wael Goravey

**Affiliations:** Department of Infectious Diseases, Communicable Diseases Centre, Hamad Medical Corporation, Doha, Qatar

A 51-year-old man, originally from India, was referred to our hospital complaining of a 2-month history of lower back pain associated with a weight loss of 4 kg. There was no significant past medical history of note, nor was there a recent history of trauma or other constitutional symptoms. Examination revealed spinal tenderness in the mid-lower back, and no neurological deficits were detected. No other findings or lymphadenopathies were observed. Laboratory tests were within normal limits except for a C-reactive protein level of 120 mg/L (range: 0–5). Magnetic resonance imaging (MRI) of the spine showed multiple osteolytic bony lesions in the cervical, thoracic, and lumbar vertebral bodies and pedicles, highly suggestive of metastasis or hematological malignancy. This finding was supported by the fluorodeoxyglucose (FDG)-positron emission tomography (PET) scan and confirmed the presence of additional lytic lesions in the bilateral ribs, clavicles, sternum, and ilium (Figure 1A). Serum tumor markers, including alpha fetoprotein, carcinoembryonic antigen, CA19-9, and prostate-specific antigen, were all within normal ranges. Computed tomography–guided fine-needle aspiration cytology was performed on the sternum lesion and the right ilium, which revealed necrotizing granulomatous inflammation and no evidence of neoplasia. However, *Mycobacterium tuberculosis* (MTB) (GeneXpert MTB/RIF; Cepheid, Sunnyvale, CA) was positive from the biopsy, and subsequently, a fully sensitive MTB was isolated. Thereafter, he was started on standard 12-month tuberculosis (TB) therapy with complete metabolic resolution of the previously seen multiple bony uptakes (Figure 1B). He had no recurrence for 12 months during follow-up.

**Figure 1.**
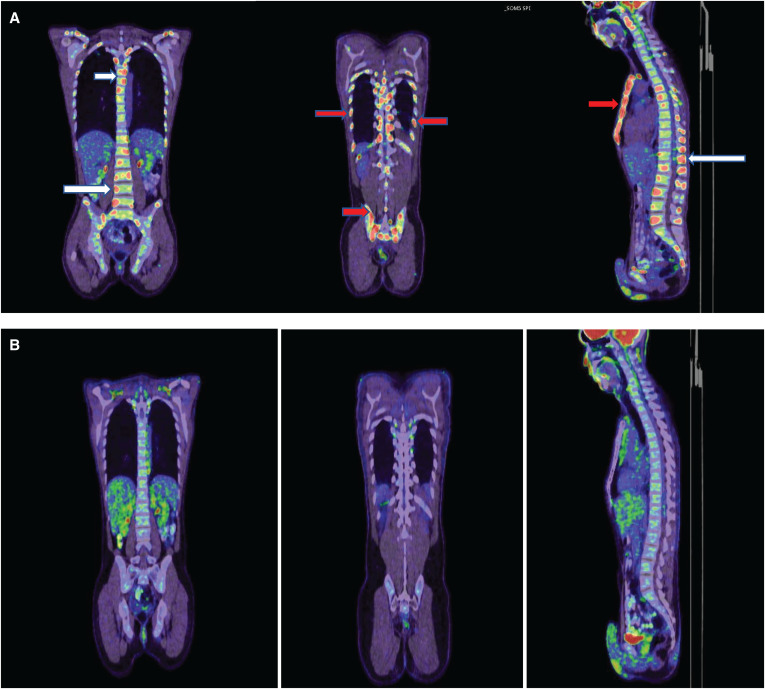
(**A**) Positron emission tomography scan demonstrating multiple osteolytic bony lesions in the cervical, thoracic, and lumbar vertebral bodies, pedicles, and spinal processes (white arrow). Also, lytic lesions involving the bilateral ribs, sternum, and ilium were noted (red arrow). (**B**) Total metabolic resolution of the lesions after 12 months of tuberculosis chemotherapy.

Extrapulmonary TB can affect all sites in the body; hence, isolated multifocal skeletal tuberculosis is not exceptional.^1^ The MRI or FDG-PET scan occurrence of multiple, noncontiguous, and posterior vertebral body involvement is rare in tuberculous spondylitis, particularly when accompanied by a paucity of symptoms and absent pulmonary involvement.^2^ Thus, they frequently elude assessment and constitute diagnostic challenges, leading to delay treatment and potentially devastating consequences.

Furthermore, active tuberculous lesions consist of epithelioid cells, Langerhans cells, and lymphocytes, which exhibit increased glucose metabolism and thus intense FDG uptake. Therefore, they can pose a diagnostic dilemma and be easily misinterpreted as metastatic or malignant lesions.^3^

A high degree of suspicion, with the aid of appropriate tests, can clarify the diagnosis and avoid morbidity and mortality. The mainstay of management is medical, and the optimal suggested treatment duration is 12–18 months.^4^

## Data Availability

The authors confirm that the datasets supporting the findings of this case are available from the corresponding author upon request.

## References

[1] Tan CH Kontoyiannis DP Viswanathan C Iyer RB, 2010. Tuberculosis: a benign impostor. AJR Am J Roentgenol 194: 555–564. 20173128 10.2214/AJR.09.3055

[2] Kumaran S Thippeswamy P Reddy B Neelakantan S Viswamitra S, 2019. An institutional review of tuberculosis spine mimics on MR imaging: cases of mistaken identity. Neurol India 67: 1408–1418. 31857525 10.4103/0028-3886.273630

[3] Ye M Huang J Wang J Ren J Tu J You W Zhu T, 2016. Multifocal musculoskeletal tuberculosis mimicking multiple bone metastases: a case report. BMC Infect Dis 16: 34. 26823075 10.1186/s12879-016-1376-7 PMC4731994

[4] Go SW Lee HY Lim CH Jee WH Wang YP Yoo IeR Kang JY, 2012. Atypical disseminated skeletal tuberculosis mimicking metastasis on PET-CT and MRI. Intern Med 51: 2961–2965. 23064577 10.2169/internalmedicine.51.8347

